# Evaluation of Metabolic Engineering Strategies on 2-Ketoisovalerate Production by Escherichia coli

**DOI:** 10.1128/aem.00976-22

**Published:** 2022-08-18

**Authors:** Li Zhou, Ying Zhu, Zhongzhe Yuan, Guangqing Liu, Zijin Sun, Shiyu Du, He Liu, Yating Li, Haili Liu, Zhemin Zhou

**Affiliations:** a The Key Laboratory of Industrial Biotechnology of Ministry of Education, School of Biotechnology, Jiangnan University, Wuxi, People’s Republic of China; North Carolina State University

**Keywords:** sustainable production of 2-ketoisovalerate, redox balance, metabolic engineering strategy, acetolactate synthase, tricarboxylic acid cycle

## Abstract

As an important metabolic intermediate, 2-ketoisovalerate has significant potential in the pharmaceutical and biofuel industries. However, a low output through microbial fermentation inhibits its industrial application. The microbial production of 2-ketoisovalerate is representative whereby redox imbalance is generated with two molecules of NADH accumulated and an extra NADPH required to produce one 2-ketoisovalerate from glucose. To achieve efficient 2-ketoisovalerate production, metabolic engineering strategies were evaluated in Escherichia coli. After deleting the competing routes, overexpressing the key enzymes for 2-ketoisovalerate production, tuning the supply of NADPH, and recycling the excess NADH through enhancing aerobic respiration, a 2-ketoisovalerate titer and yield of 46.4 g/L and 0.644 mol/mol glucose, respectively, were achieved. To reduce the main by-product of isobutanol, the activity and expression of acetolactate synthase were modified. Additionally, a protein degradation tag was fused to pyruvate dehydrogenase (PDH) to curtail the conversion of pyruvate precursor into acetyl-CoA and the generation of NADH. The resulting strain, 050TY/pCTSDTQ487S-RBS55, was initially incubated under aerobic conditions to attain sufficient cell mass and then transferred to a microaerobic condition to degrade PDH and inhibit the remaining activity of PDH. Intracellular redox imbalance was relieved with titer, productivity and yield of 2-ketoisovalerate improved to 55.8 g/L, 2.14 g/L h and 0.852 mol/mol glucose. These results revealed metabolic engineering strategies for the production of a redox-imbalanced fermentative metabolite with high titer, productivity, and yield.

**IMPORTANCE** An efficient microbial strain was constructed for 2-ketoisovalerate synthesis. The positive effect of the *leuA* deletion on 2-ketoisovalerate production was found. An optimal combination of overexpressing the target genes was obtained by adjusting the positions of the multiple enzymes on the plasmid frame and the presence of terminators, which could also be useful for the production of downstream products such as isobutanol and l-valine. Reducing the isobutanol by-product by engineering the acetolactate synthase called for special attention to decreasing the promiscuous activity of the enzymes involved. Redox-balancing strategies such as tuning the expression of the chromosomal pyridine nucleotide transhydrogenase, recycling NADH under aerobic cultivation, switching off PDH by degradation, and inhibiting the expression and activity under microaerobic conditions were proven effective for improving 2-ketoisovalerate production. The degradation of PDH and inhibiting this enzyme's expression would serve as a means to generate a wide range of products from pyruvate.

## INTRODUCTION

So far, the metabolic intermediate 2-Ketoisovalerate has been mainly applied in drugs that treat chronic kidney disease (1, 2) and uremic hyperphosphatemia (3). Furthermore, as the precursor of branched-chain amino acids and other important chemicals, such as pantothenate, coenzyme A, glucosinolate (4), and isobutanol (5), it can also be used for the production of these pharmaceutical agents and biofuels (6). A variety of chemical synthesis methods have been applied to produce 2-ketoisovalerate (7). However, the process requires nonrenewable resources, and it suffers from harsh conditions and toxic by-products. The production using renewable resources such as glucose, which is the hydrolysis product of starch, with microbial fermentation, would be a more environmentally friendly and sustainable alternative. 2-Ketoisovalerate is generally reported as a metabolic intermediate of isobutanol fermentation and is generally minimized during the production of isobutanol (5, 8). To the best of our knowledge, only Corynebacterium glutamicum has been engineered to produce 35 g/L 2-ketoisovalerate as the main target product under aerobic cultivation (6, 9). A more efficient microbial cell factory for 2-ketoisovalerate production would promote its industrial application.

The ultimate aim of any fermentation process is to achieve high titer, yield, and productivity (10), which are determined by the cellular substance and energy metabolisms. To achieve high yield, cellular substance metabolism should be adapted to maximize the metabolic flux for the target product by deleting competing routes, overexpressing target routes (11), and controlling biomass accumulation, which competes for substrate precursors with the target product (12). Furthermore, special attention should be paid to the promiscuous activity of the enzymes involved. For example, in addition to condensing two molecules of pyruvate to form 2-acetolactate, acetolactate synthase (AlsS) could also catalyze the decarboxylation of 2-ketoisovalerate to generate isobutyraldehyde, which is then converted to isobutanol via intracellular alcohol dehydrogenase (5). The accumulation of isobutanol using 2-ketoisovalerate as a precursor could substantially reduce 2-ketoisovalerate yield and be toxic to the cell. Molecular modification of AlsS to diminish the decarboxylase activity but maintain the synthase activity would solve the problem.

**FIG 1:**
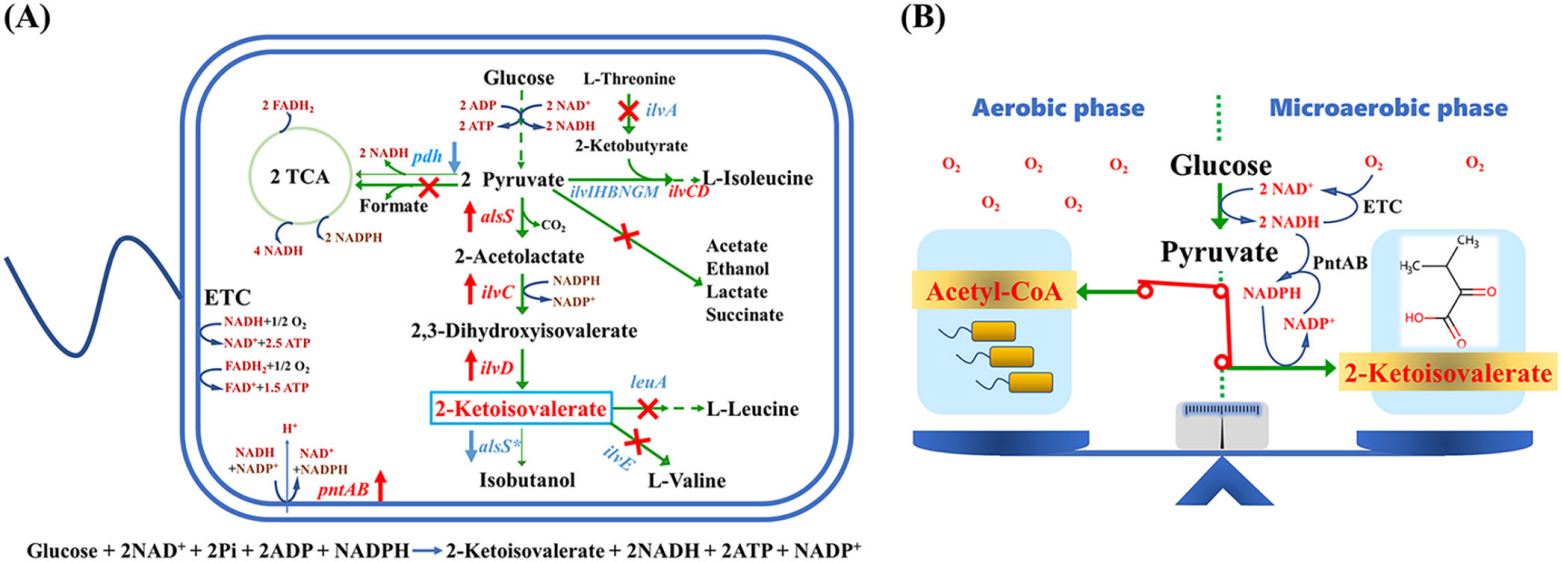
Metabolic engineering strategies applied in the present study. (A) Regulation of metabolic pathways for 2-ketoisovalerate production in E. coli. The overexpressed enzymes are indicated by red arrows, and the deleted competing routes are indicated by red crosses. Genes and enzymes include *alsS*, Bacillus subtilis-derived acetolactate synthase; *ilvC*, E. coli-derived acetohydroxy acid isomeroreductase; *ilvD*, E. coli-derived dihydroxy acid dehydratase; *pntAB*, pyridine nucleotide transhydrogenase; *ilvA*, threonine deaminase; *ilvIHBNGM*, acetolactate synthase; *ilvE*, branched-chain amino acid aminotransferase; *leuA*, 2-isopropylmalate synthase; and *pdh*, pyruvate dehydrogenase. TCA, tricarboxylic acid cycle; ETC, electron transport chain. (B) Redox-balancing strategies for 2-keotisovalerate production in E. coli.

Regulating the circulation of electron carrier NAD^+^ affects not only the distribution of metabolic flux but also cellular energy metabolism, cell activity, and productivity of the target chemicals (13). Notably, the synthesis of 2-ketoisovalerate from glucose generates imbalanced redox status whereby two molecules of NADH are accumulated and an extra molecule of NADPH is required (Fig. 1A). Therefore, generating reducing force in the form of NADPH from the excess NADH would effectively enhance 2-ketoisovalerate production. Although transhydrogenase has been used to promote this catalysis (14–17), fine-tuning the strength of this conversion for the efficient production of chemicals has rarely been reported. To recycle the overgenerated NADH and attain redox balance, sparging air into the culture broth to provide O_2_ as the final electron acceptor of the electron transport chain (ETC) is a convenient and effective way. Providing more O_2_ than required for redox balance could obtain higher volumetric productivity of the target fermentative product (18), whereas the O_2_ supply should be controlled at lower concentrations to prevent reducing force depletion and an impairment in product yield (19). Conversely, the conversion of pyruvate to acetyl-CoA by pyruvate dehydrogenase (PDH) and the following tricarboxylic acid (TCA) cycle could intensify redox imbalance with the generation of a large amount of NADH and compete with the production of 2-ketoisovalerate for the same metabolic precursor of pyruvate. A complete deletion of PDH could significantly improve the yield of 2-ketoisovalerate to 0.47 mol/mol glucose in C. glutamicum. The mutant was auxotrophic for acetate as an additional carbon source (6). Another approach to avoiding this auxotrophy would merely switch off the PDH after accumulating an adequate amount of cell mass. Therefore, it would be better to effectively switch off the PDH for efficient 2-ketoisovalerate production after accumulating an adequate amount of cell mass.

In the present study, several metabolic engineering strategies (Fig. 1A) were applied to produce 2-ketoisovalerate in Escherichia coli, which is a widely used cell factory. Deleting the competing routes, overexpressing the key enzymes for 2-ketoisovalerate production, and minimizing the promiscuous activity of the acetolactate synthase regulated the intracellular substance metabolism and maximized the metabolic flux for 2-ketoisovalerate production. Tuning the supply of NADPH and limiting the PDH activity through regulating O_2_ supply and degrading PDH were combined to relieve the redox imbalance of the 2-ketoisovalerate-producing strain. After obtaining sufficient cell mass under aerobic cultivation, 2-ketoisovalerate production was switched on, and the PDH was switched off under microaerobic conditions to achieve high 2-ketoisovalerate yield, titer, and productivity (Fig. 1B). The metabolic engineering strategies applied in this study may be useful for the production of other chemicals that generate imbalanced redox homeostasis.

## RESULTS AND DISCUSSION

### Effects of deleting the competing routes and overexpressing the target routes.

Deleting the competitive routes and overexpressing the target routes are cardinal strategies and have been applied successfully in industrial fermentation (11, 20). However, systematically optimizing the combination of gene deletions and overexpression remains challenging. To maximize metabolic flux for 2-ketoisovalerate production and to construct a chassis strain, the combination of competing route deletions and target route overexpressions were optimized in the present investigation. Strain E. coli B0016-050 (*ack-pta pflB adhE frdA ldhA*) (21), harboring the deletions which curtail the formation of acetate, formate, ethanol, succinate, and lactate, was used as the starting strain for 2-ketoisovalerate production. In addition, the 2-ketoisovalerate-utilizing route *ilvE* (encoding branched-chain amino acid aminotransferase) in B0016-050 was deleted by a simultaneous insertion of the T7 RNA polymerase gene to generate strain 050T. The 2-ketoisovalerate-competing route *ilvA* (encoding threonine deaminase) and -consuming route *leuA* (encoding 2-isopropylmalate synthase) (Fig. 1A) were further deleted in 050T to produce strains 050T1, 050T2, and 050T3, respectively. Three key genes, *alsS*, *ilvC*, and *ilvD* (encoding Bacillus subtilis-derived acetolactate synthase, E. coli-derived acetohydroxy acid isomeroreductase, and dihydroxy acid dehydratase, respectively) were overexpressed by using plasmid pCTSDT to enhance the synthesis of 2-ketoisovalerate in the gene-deleted strains.

**FIG 2:**
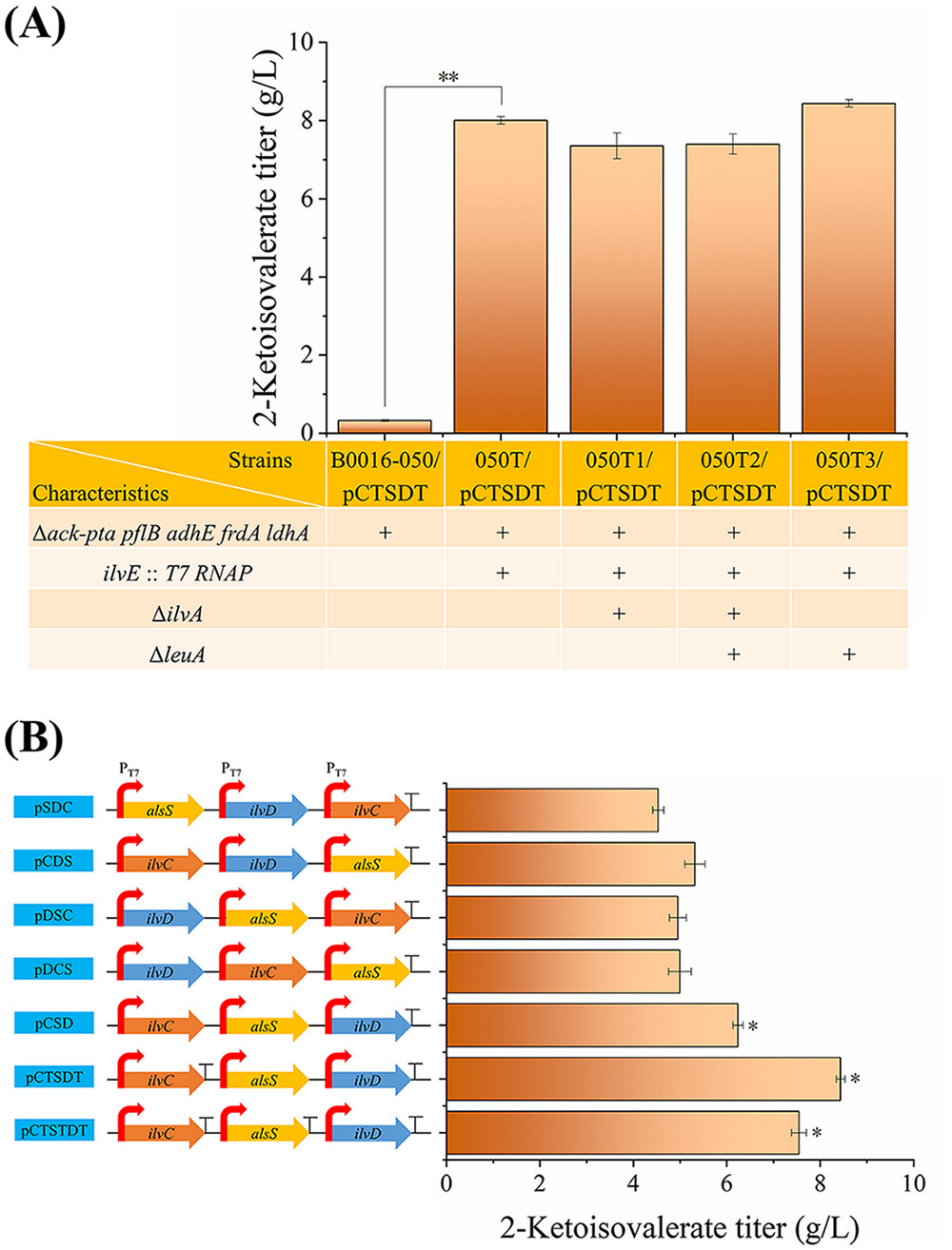
Comparison of 2-ketoisovalerate production by strains with gene deletion and overexpression. (A) Comparison of 2-ketoisovalerate synthesis by strains with combined gene deletions. (B) Comparison of 2-ketoisovalerate synthesis by strain 050T3 with different plasmids. T-shaped bar indicates a T7 transcription terminator. When the cell concentration reached an OD_600_ of 0.8, cells were induced by adding IPTG. Shaking speed was maintained at 200 rpm during the entire fermentation process. Fermentations were stopped at 26 h.

As shown in Fig. 2, the starting strain B0016-050/pCTSDT produced 0.33 g/L 2-ketoisovalerate. These data significantly improved to 8.01 g/L using strain 050T/pCTSDT, indicating that the integrated T7 RNA polymerase on the 050T chromosome functioned well in the transcription of the T7 promoter-targeted *BsalsS*, *EcilvC*, and *EcilvD* genes for 2-ketoisovalerate synthesis. As previously reported, production of l-valine, a downstream product of 2-ketoisovalerate, could be increased by downmodulating *ilvA* expression or deleting *ilvA* (22) to block 2-ketobutyrate supply for the competitive utilization of pyruvate by the native acetolactate synthase (encoded by *ilvIH*, *ilvBN*, and *ilvGM* for l-isoleucine formation [23]) (Fig. 1A). However, the complete inactivation of the *ilvA* gene could not further enhance 2-ketoisovalerate production in strains 050T1/pCTSDT and 050T2/pCTSDT herein. This implied that the native acetolactate synthases were not as efficient as the overexpressed AlsS in this study for pyruvate assimilation; otherwise, the l-isoleucine auxotrophy generated by the deletion of *ilvA* would impair the metabolic balance for 2-ketoisovalerate production. As a result, the *ilvA* gene deletion was not involved in the following strains. The deletion of another 2-ketoisovalerate-consuming route encoded by the *leuA* gene in strain 050T/pCTSDT produced 050T3/pCTSDT, and 2-ketoisovalerate production was further improved by 5%, which was also consistent with the *leuA* deletion effect for the improvement of l-valine accumulation (24). Consequently, strain 050T3 exhibited the optimal gene deletions for 2-ketoisovalerate production among the above-described strains. Compared to strain E. coli BL21(DE3)/pCTSDT, which generated only 1.7 g/L 2-ketoisovalerate without the inactivation of competing routes (25), 2-ketoisovalerate production was 5 times higher in 050T3/pCTSDT. These results suggest the significance of deleting the relevant competing routes. Although deletions of the acetate-generating route and pyruvate-consuming route have been reported for the production of 2-ketoisovalerate (6, 9), more gene deletions on competitive routes, such as the synthetic routes for formate, lactate, succinate, and ethanol, were involved in the present study, and they resulted in a higher 2-ketoisovalerate yield. The deletion of *ilvE* is normally included in strains for 2-ketoisovalerate and isobutanol production (6, 9, 26). However, the effects of *ilvA* and *leuA* deletions on 2-ketoisovalerate or isobutanol production have not been investigated, and a positive *leuA* deletion was identified in the present study.

AlsS, IlvC, and IlvD are essential for 2-ketoisovalerate synthesis. Overexpression of *BsalsS*, *EcilvC*, and *EcilvD* genes was optimized by altering their positions on the plasmid frame and adding terminators after every gene. The recombinant plasmids were tested for 2-ketoisovalerate synthesis in strain 050T3 (Fig. 2B). After altering the positions of genes on the plasmid frame, the 2-ketoisovalerate titer was improved by 38% by using plasmid pCSD. Considering that every gene was controlled under a separately strong T7 promoter, the order of the genes may have an effect on expression by read-through transcription. Furthermore, failure to terminate transcription efficiently causes problems in plasmid-based genetic devices due to read-through into the plasmid origin of replication (27). Correspondingly, the addition of a terminator after the *ilvC* gene significantly improved the 2-ketoisovalerate titer to 8.44 g/L. However, adding terminators after every gene could not further improve the 2-ketoisovalerate titer, indicating that stopping transcription at a defined point is essential for a robust design. Plasmid pCTSDT was optimal for 2-ketoisovalerate synthesis *in vivo*, and the 2-ketoisovalerate production was improved by 86% compared to that of the original plasmid, pSCD. The improved 2-ketoisovalerate titers were consistent with the increased expression of the three essential enzymes and the catalytic ability for 2-ketoisovalerate synthesis from pyruvate *in vitro* (25). These results indicated that engineering the order of genes and terminators are effective strategies for optimizing protein expression and the resulting catalytic efficiency. The optimized combinations of gene deletion and overexpression here could also be used for the production of downstream products, such as isobutanol and l-valine.

### Effects of tuning the circulation of cofactors.

**(i) Overexpression of pyridine nucleotide transhydrogenase.** The synthesis of one molecule of 2-ketoisovalerate from glucose leads to redox imbalance with the net accumulation of two molecules of NADH and an extra requirement of one molecule of NADPH (Fig. 1A). Although an alternative ketol-acid reductoisomerase (IlvC) mutant has been engineered to be NADH dependent to avoid NADPH requirement, the enzymatic activity is significantly lower (14) and was not involved in the present study. The excess NADH that is generated during 2-ketoisovalerate synthesis should be recycled through the ETC to achieve redox homeostasis, and therefore, producing the reduced coenzyme through converting to NADPH, which could not be utilized by the ETC, may save the reducing force pool for 2-ketoisovalerate production against aerobic respiration. Consequently, the conversion of NADH to NADPH was intensified to compensate for the insufficiency of NADPH in this study.

**FIG 3:**
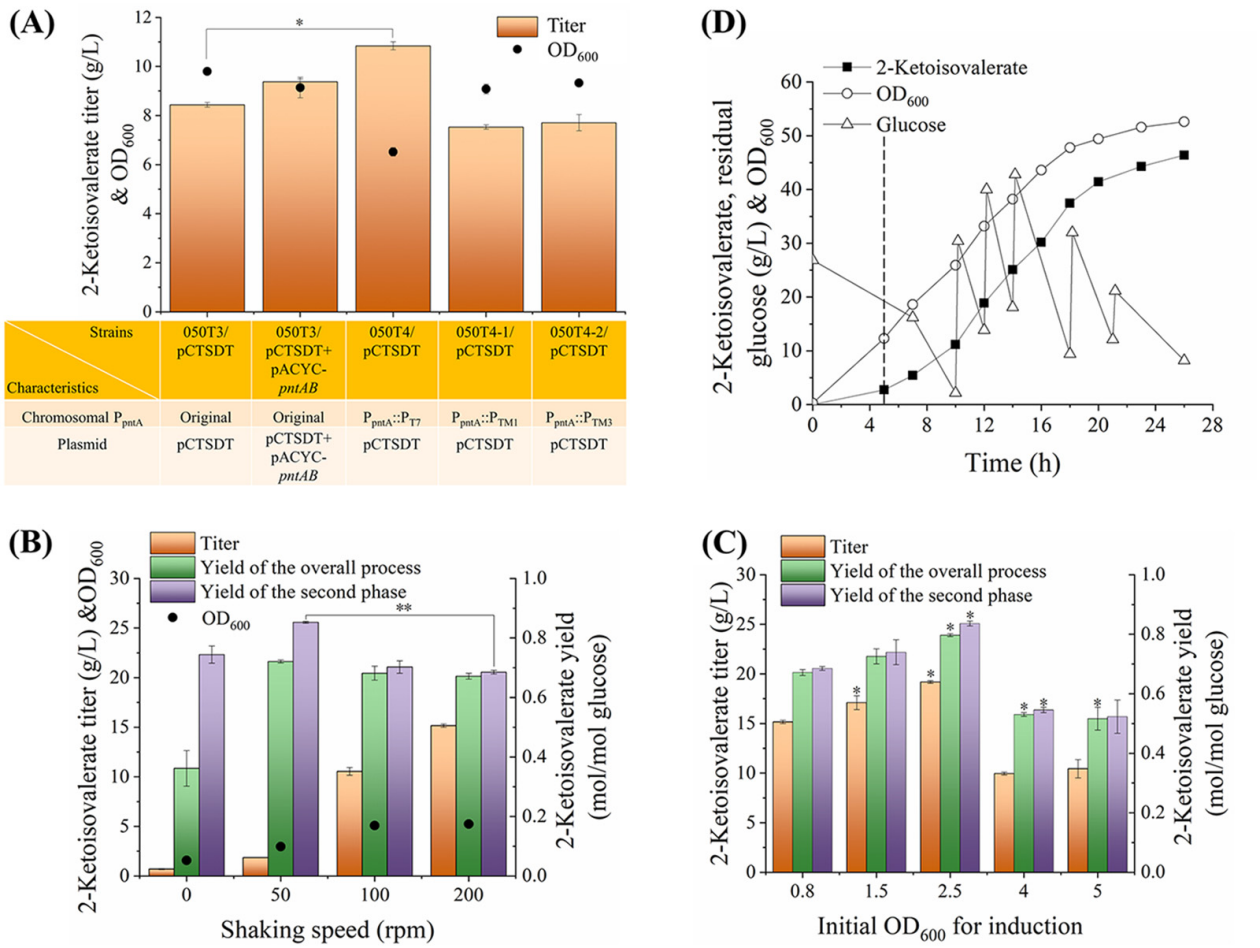
Effects of regulating cofactors on production of 2-ketoisovalerate. (A) Comparison of 2-ketoisovalerate synthesis by strains overexpressing the *pntAB* gene. When the cell concentration reached an OD_600_ of 0.8, cells were induced by adding IPTG. Shaking speed was maintained at 200 rpm during the entire fermentation process. Fermentations were stopped at 26 h. (B) Optimization of the shaking speed in shake-flask experiments using strain 050T4/pCTSDT. Fermentations were stopped at 36 h. (C) Optimization of the transition time point from the cell growth phase to the 2-ketoisovalerate production phase using strain 050T4/pCTSDT. (D) Representative experiment showing the production of 2-ketoisovalerate by strain 050T4/pCTSDT in a 5-L bioreactor. When the cell density reached an OD_600_ of approximately 12, 2-ketoisovalerate production phase was initiated (indicated by the dotted line). Dissolved O_2_ concentration was maintained above 30% saturation in a cascade by stirring at 200 to 1,000 rpm and sparging air into the bioreactor at 3 to 10 L/min during the entire fermentation process. Additions of glucose of 60, 60, 60, 60, and 30 g were made to the bioreactor to maintain the residual glucose concentration above 10 g/L.

The E. coli-derived pyridine nucleotide transhydrogenase (encoded by *pntAB*), which is responsible for catalyzing NADH to NADPH, was overexpressed under the transcriptional initiation of the T7 promoter and its derivatives. As shown in Fig. 3A, 2-ketoisovalerate synthesis was improved by 11% after the overexpression of this transhydrogenase on a separate plasmid under the T7 promoter, implying the effectiveness of intensifying the NADPH supply. This result was also consistent with the promotional role of PntAB on the production of other NADPH-dependent metabolites (14–16). Although the extensive formation of NADPH could be spontaneously counterbalanced by the transhydrogenase UdhA (28), tuning the expression of PntAB could avoid unnecessary energy-consuming futile circulation. Consequently, the T7 promoter, as well as TM1 and TM3 promoters, with transcriptional strengths of 92% and 16% of that of the T7 promoter (29), respectively, were used to substitute the promoter of the chromosomal *pntAB* gene. With a stable integration of the T7 promoter before the chromosomal *pntAB* gene, 2-ketoisovalerate production in the resulting strain 050T4/pCTSDT was further enhanced by 16% in contrast to strain 050T3/pCTSDT+pACYC-pntAB. Therefore, the expression of one copy of the *pntAB* gene under the T7 promoter was sufficient for 2-ketoisovalerate fermentation, and a decrease in metabolic burden by removing the plasmid would also contribute to the improved titer (30). Meanwhile, the activities of PntAB and UdhA (see Fig. S1A in the supplemental material) were significantly increased in strains 050T3/pCTSDT+pACYC-pntAB and 050T4/pCTSDT, implying the higher coenzyme-adjusting abilities. The coordination between PntAB and UdhA resulted in a significantly higher intracellular NADPH/NADP^+^ ratio in strain 050T4/pCTSDT (Fig. S1B), which was also consistent with the production of 2-ketoisovalerate and indicated the effectiveness of tuning the expression of PntAB. In contrast, 2-ketoisovalerate synthesis and the NADPH/NADP^+^ ratio could not be improved with TM1 and TM3 promoters in the resulting strains 050T4-1/pCTSDT and 050T4-2/pCTSDT, which might be because of neutral adjustment of the cell, inappropriate gene structures, or insufficient promoting strengths. Therefore, strain 050T4/pCTSDT was a more efficient 2-ketoisovalerate producer for the aerobic production of 2-ketoisovalerate.

Transhydrogenase has been used to supplement NADPH deficiency from NADH (14–17). However, the strength of this conversion has not been tuned for the optimal production of chemicals. In the present investigation, the highest 2-ketoisovalerate titer was reached while tuning the expression of the chromosomal *pntAB* gene with the strong T7 promoter rather than other weaker promoters or overexpression on the plasmid. This result supported the conclusion that tuning the desired enzymes to moderate rather than high expression levels could achieve an overproduction of the desired biochemicals (31). The expression intensity of PntAB would be instructive for the production of other NADPH-deficient metabolites.

**(ii) Optimization of fermentation conditions of strain 050T4/pCTSDT.** In the present study, a two-phase fermentation process with a separate cell growth phase and 2-ketoisovalerate production phase was applied to achieve high productivity after accumulating sufficient cell mass. During the 2-ketoisovalerate production phase, the ETC was used to recycle the overaccumulated reducing force, with O_2_ as the ultimate electron acceptor (Fig. 1). Therefore, the effect of shaking speed, which is the main factor affecting dissolved O_2_ in shake-flask cultivations on 2-ketoisovalerate production, was investigated. As shown in Fig. 3B, strain 050T4/pCTSDT produced higher 2-ketoisovalerate at a higher shaking speed. The highest titer of 15.2 g/L was achieved at 200 rpm during 36 h fermentation, suggesting an efficient circulation of the reducing coenzymes. In contrast, the highest 2-ketoisovalerate yield was realized at 50 rpm, with an overall yield of 0.721 mol/mol glucose and a second-phase yield of 0.853 mol/mol glucose, which were 7% and 24% higher than that at 200 rpm, respectively, implying that the conversion of pyruvate to acetyl-CoA competed with the conversion of pyruvate to 2-ketoisovalerate under the more oxygenated conditions at 200 rpm than at 50 rpm. However, the yield was significantly lower than the theoretical value of 1 mol/mol glucose with substantial cell mass accumulation, even under O_2_-limited conditions. 2-Ketoisovalerate production and cell growth phenotype at the shaking speed of 100 rpm implied a possible option for synchronizing high 2-ketoisovalerate titer, productivity, and yield under microaerobic conditions. Considering the significantly higher 2-ketoisovalerate titer, 200 rpm was selected for aerobic 2-ketoisovalerate production. The transition time point from the cell growth phase to the 2-ketoisovalerate production phase would affect the carbon flow between the cell mass and 2-ketoisovalerate synthesis and was further optimized. As shown in Fig. 3C, this transition time point significantly affected 2-ketoisovalerate production. The highest 2-ketoisovalerate titer of 19.2 g/L, overall yield of 0.797 mol/mol glucose, and second-phase yield of 0.836 mol/mol glucose were achieved while switching to the second phase after cell density reached an optical density at 600 nm (OD_600_) of 2.5. Consequently, incubation with shaking at 200 rpm and induction at an OD_600_ of 2.5 were the optimal fermentation conditions for the shake-flask experiment using strain 050T4/pCTSDT.

These results implied that after deleting the pyruvate formate lyase (encoded by *pfl*), which is responsible for anaerobic catalysis of acetyl-CoA from pyruvate (32), the remaining PDH complex (encoded by *aceEF* and *lpd*) in strain 050T4/pCTSDT could still function even under microaerobic conditions, and this was consistent with a previous report (33). A large amount of reducing power is generated by PDH and the following TCA cycle, which should be reoxidized through the ETC with O_2_ as the ultimate electron acceptor to achieve intracellular redox balance (Fig. 1A). Although the highest 2-ketoisovalerate yield was achieved under microaerobic conditions, we hypothesized that the fluxes of NADH synthesis and the ETC would be inconsistent for achieving redox balance, resulting in a significantly lower 2-ketoisovalerate titer. Genetically regulating the PDH activity to lower the NADH synthesis would be helpful for simultaneously achieving the highest 2-ketoisovalerate yield and titer under microaerobic cultivation.

**FIG 4:**
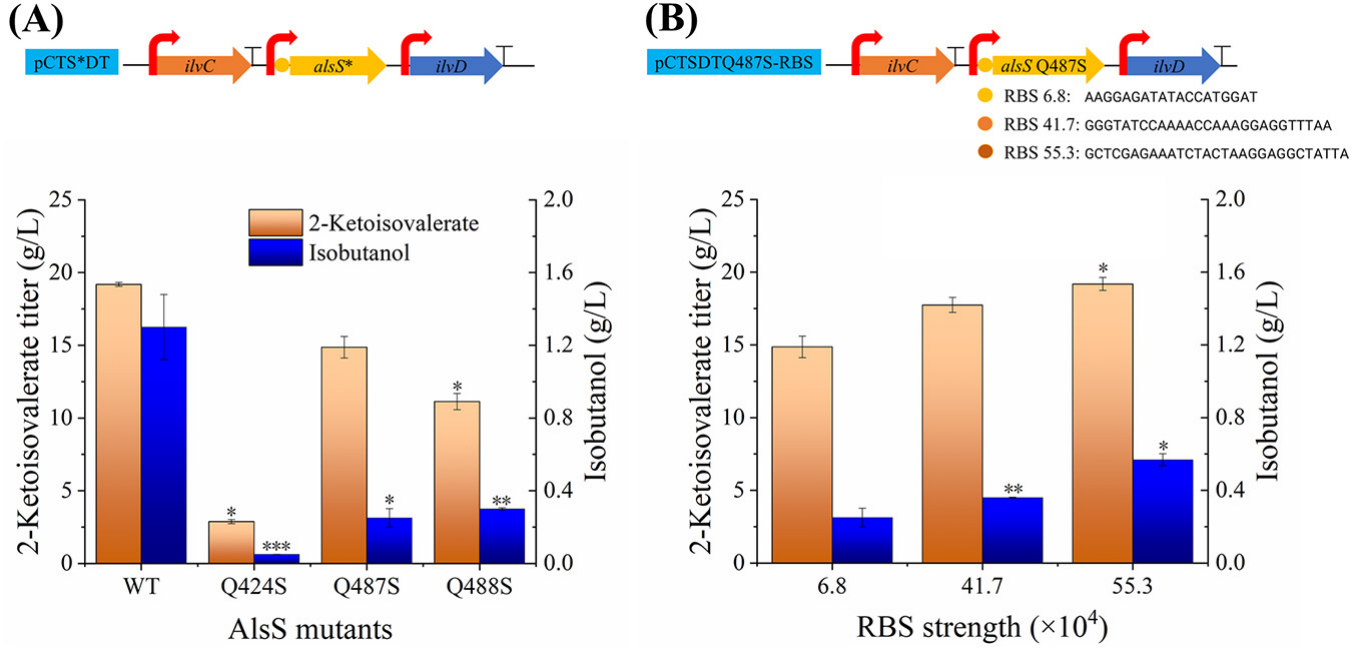
Comparison of fermentation data of strains with engineered AlsS (A) and RBS (B). When the cell concentration reached an OD_600_ of 2.5, IPTG was added. The shaking speed was maintained at 200 rpm during the entire fermentation process. Fermentations were stopped at 36 h.

**(iii) 2-Ketoisovalerate fermentation in 5-L bioreactor using strain 050T4/pCTSDT.** Fermentation of 2-ketoisovalerate using strain 050T4/pCTSDT was further carried out in a 5-L bioreactor to test the effect of the combined engineering strategies (deletion of competing route, overexpression of target route, and tuning cofactor circulation) under controlled production conditions (Fig. 3D). A 2-ketoisovalerate titer of 46.4 g/L, with a volumetric productivity of 1.78 g/L h (Table 1), was reached. These levels were 2.4- and 3.3-fold larger than that of the shake-flask experiment (Fig. 3C), indicating the benefit of enlarged fermentation. However, the 2-ketoisovalerate yield during the overall fermentation process and the second process were determined as 0.644 and 0.657 mol/mol glucose, respectively, which were significantly lower than that of the shake-flask experiment (Fig. 3C). The accumulation of 13.1 g/L isobutanol with a second-phase yield of 0.284 mol/mol glucose (Table 1) and the significantly higher cell mass accumulation in the bioreactor were partially responsible for the lower 2-ketoisovalerate yield. The high level of isobutanol would be generated by AlsS (from Bacillus subtilis), which has a side activity of decarboxylating 2-ketoisovalerate to isobutanol in addition to its main activity of acetolactate synthase (5). The higher *K_m_* value of AlsS, with 2-ketoisovalerate as the substrate (5), meant that the synthesis of isobutanol was significantly greater at a higher 2-ketoisovalerate titer in enlarged cultivation than in the shake-flask experiment (Fig. 4A), serving as the main by-product and toxicity to the strain. Therefore, AlsS should be modified to minimize isobutanol accumulation and improve the 2-ketoisovalerate yield. The TCA cycle competes for the pyruvate precursor, the regulation of which would also be beneficial for improving 2-ketoisovalerate yield.

**TABLE 1:** Comparison of fermentation data from the bioreactor experiments

Strain	Relevant genotype	Data for 2-ketoisovalerate					By-product yield (mol/mol glucose)^*d*^ of:				Source or reference
		Titer (g/L)	Volumetric productivity (g/L h)^*a*^	Specific productivity (g/g h)^*b*^	Specific glucose consumption rate (g/g h)^*c*^	Yield (mol/mol glucose)^*d*^^,^^*e*^	Isobutanol	Pyruvate	Succinate	Acetate	
E. coli 050T4/pCTSDT	Δ*ack-pta*, Δ*pflB*, Δ*adhE*, Δ*frdA*, Δ*ldhA*, Δ*leuA*, *ilvE*::*T7RNAP*, P_pntA_::P_T7_, pETDuet plasmid harboring P_T7_-*ilvC*-T7 terminator-P_T7_-*alsS*-P_T7_-*ilvD*	46.4 ± 1.8	1.78 ± 0.06	0.112 ± 0.004	0.266 ± 0.013	0.644 ± 0.023^*d*^, 0.657 ± 0.032^*e*^	0.284 ± 0.009	0.016 ± 0.001	0.119 ± 0.004	0.075 ± 0.003	This study
E. coli 050TY/pCTSDTQ487S-RBS55	Δ*ack-pta*, Δ*pflB*, Δ*adhE*, Δ*frdA*, Δ*ldhA*, Δ*leuA*, *ilvE*:: *T7RNAP*, P_pntA_:: P_T7_, *aceF*-DAS+4 tag, pETDuet plasmid harboring P_T7_-*ilvC*-T7 terminator-P_T7_-RBS55-*alsS*Q424S-P_T7_-*ilvD*	55.8 ± 2.1	2.14 ± 0.07	0.162 ± 0.011	0.253 ± 0.012	0.852 ± 0.034^*d*^, 0.992 ± 0.043^*e*^	0.036 ± 0.001	0.022 ± 0.001	0.040 ± 0.002	0.134 ± 0.006	This study
C. glutamicum Δ*aceE* Δ*pqo* Δ*ilvE* (pJC4ilvBNCD)	Δ*aceE*, Δ*pqo*, Δ*ilvE*, pJC4ilvBNCD plasmid harboring *ilvBNCD*	21.8 ± 3.2	0.53 ± 0.07	Not reported	Not reported	0.47 ± 0.05^*d*^	Not reported	Not reported	Not reported	Not reported	6
C. glutamicum *aceE* A16 Δ*pqo* Δ*ppc* Δ*ilvE* (pJC4ilvBNCD)	P_aceE_::P_A16_, Δ*pqo*, Δ*ppc*, Δ*ilvE*, pJC4ilvBNCD plasmid harboring *ilvBNCD*	35	0.8	Not reported	Not reported	0.24^*d*^	Not reported	Not reported	Not reported	Not reported	9

a Average volumetric productivity for 2-ketoisovalerate during the overall fermentation process.

b Average specific productivity for 2-ketoisovalerate during the second fermentation phase.

c Average specific glucose consumption rate during the second fermentation phase.

d Average yield for product during the overall fermentation process.

e Average yield for 2-ketoisovalerate during the second fermentation phase.

### Minimizing the accumulation of isobutanol.

**(i) Molecular modification of AlsS to minimize the synthesis of isobutanol.** Isobutanol is potentially toxic to microorganisms. As tested by the host strain E. coli 050T4, cell growth was substantially inhibited with the supplementation of higher than 1.5 g/L isobutanol (Fig. S2). Both final cell densities (Fig. S2A) and growth rates (Fig. S2B) were correspondingly reduced with increased supplemented isobutanol. Therefore, the high accumulation of isobutanol in large-scale fermentation (Fig. 3D) should be minimized to attain optimal cell growth properties and higher 2-ketoisovalerate production.

In addition to condensing two pyruvate molecules to acetolactate, AlsS could catalyze the conversion of 2-ketoisovalerate into isobutyraldehyde, which could then be reduced to isobutanol (34). The Q424 and Q487 sites of AlsS are important for the recognition and positioning of the second incoming pyruvate molecule (34), and the Q488 adjacent to the Q487 site would function similarly. Therefore, the Q424S, Q487S, and Q488S mutants of AlsS were, respectively, constructed on the pCTSDT plasmid to minimize the accumulation of isobutanol. Although the activities of condensing two pyruvate molecules using the Q424S and Q487S mutants were higher than those using the wild-type AlsS (34), 2-ketoisovalerate production was lower using these mutants during the fermentation processes (Fig. 4A). This could be because these mutants had altered catalytic activities under the lower cultivation temperature used in this study. Conversely, isobutanol accumulation using the Q424S and Q487S mutants was substantially lower than the wild-type AlsS (Fig. 4A), although higher than 53% of activity with 2-ketoisovalerate as the substrate remained (34). The Q488S mutant showed similar alterations in products to that of the Q487S mutant (Fig. 4A), indicating a similar catalytic function of these two amino acid sites. Strain 050T4/pCTSDTQ487S retained 78% 2-ketoisovalerate titer compared to that of strain 050T4/pCTSDT, and the isobutanol accumulation decreased by 81%. In light of the potential for minimizing isobutanol accumulation while maintaining 2-ketoisovalerate production, strain 050T4/pCTSDTQ487S was used for further study.

**(ii) Engineering the ribosome binding site of AlsS for 2-ketoisovalerate production.** To maximize the 2-ketoisovalerate production of strain 050T4/pCTSDTQ487S, the expression level of AlsSQ487S was tuned by engineering its ribosome binding site (RBS) (Fig. 4B). RBS sequences designed by the RBS calculator (version 2.1) (35) were used to substitute the original RBS of the plasmid backbone. With increased RBS strength, the production of 2-ketoisovalerate improved. At the highest RBS strength (55.3 × 10^4^ Au) predicted by the RBS calculator, a 2-ketoisovalerate titer of 19.2 g/L was achieved in strain 050T4/pCTSDTQ487S-RBS55, which reached the same level as that of strain 050T4/pCTSDT. Although the accumulation of isobutanol in strain 050T4/pCTSDTQ487S-RBS55 increased to 0.57 g/L, this by-product was also 56% lower than that of strain 050T4/pCTSDT, thus suggesting successful minimization of isobutanol without impairing 2-ketoisovalerate production.

As an isoenzyme of AlsS, acetohydroxyacid synthase (AHAS) has been used for 2-ketoisovalerate production in Corynebacterium glutamicum without producing isobutanol as a by-product (6). However, AHAS is also involved in the synthesis of 2-aceto-2-hydroxybutyrate in addition to 2-acetolactate and is feedback inhibited by 2-ketoisovalerate and branched-chain amino acids, resulting in a low 2-ketoisovalerate titer (6). Alternatively, AlsS from B. subtilis generates 2-acetolactate but not 2-aceto-2-hydroxybutyrate (24), which could reduce the accumulation of l-isoleucine as a by-product (Fig. 1A). AlsS could not be affected by the feedback inhibition of branched-chain amino acids (36). Although AlsS has 2-ketoisovalerate decarboxylase activity, we minimized this activity and maintained the overall acetolactate synthase activity through molecular modification and RBS optimization, respectively. The isobutanol by-product was successfully minimized without decreasing the 2-ketoisovalerate titer (Fig. 4), which would benefit the large-scale production of 2-ketoisovalerate.

### Effects of switching the pyruvate dehydrogenase.

**FIG 5:**
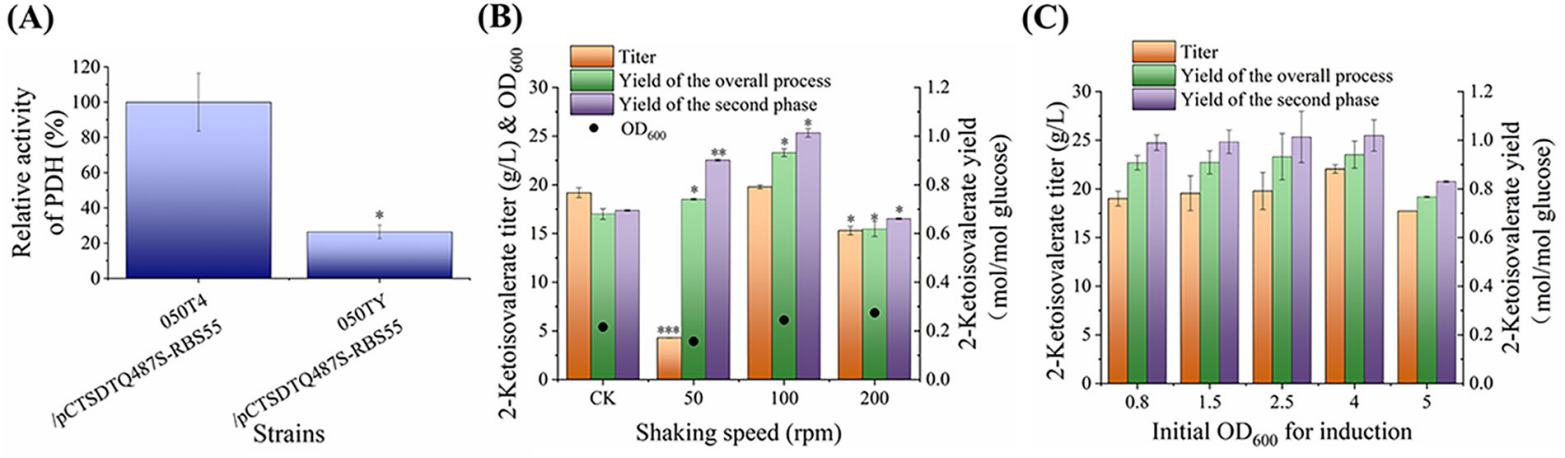
Comparison of fermentation data of strains with weakened TCA cycle. (A) Comparison of enzymatic activity of PDH. Strains were cultured at 200 rpm for 12 h. (B) Effect of shaking speed on strain 050TY/pCTSDTQ487S-RBS55. When the cell concentration reached an OD_600_ of 2.5, cells were shifted from the cell growth phase to the 2-ketoisovalerate production phase by adding IPTG and the indicated shaking speed. Fermentations were stopped at 36 h. CK indicates the experiment using strain 050T4/pCTSDTQ487S-RBS55 at 200 rpm. (C) Effect of transition time point between cell growth and 2-ketoisovalerate production phases on strain 050TY/pCTSDTQ487S-RBS55. The 2-ketoisovalerate production phase was initiated by adding IPTG. Shaking speed was maintained at 200 rpm during the initial cell growth phase and at 100 rpm during the 2-ketoisovalerate production process. Fermentations were stopped at 36 h.

The conversion of pyruvate to acetyl-CoA by pyruvate dehydrogenase (PDH) competes with 2-ketoisovalerate synthesis for the pyruvate precursor. The complete deletion of PDH will block cell growth on glucose as the sole carbon source. To achieve higher 2-ketoisovalerate production under microaerobic conditions while not considerably reducing the growth capacity of the strain, PDH was knocked down using a relatively weak DAS+4 degradation tag (37). This tag could effectively guide the degradation of the target protein in strain 050T4 (Fig. S3) and was fused to the C terminus of the AceF subunit of the PDH complex on the chromosome of strain 050T4 to create strain 050TY. As shown in Fig. 5A, the PDH activity of strain 050TY was significantly lower than that of strain 050T4, indicating success in regulating PDH.

Strain 050TY/pCTSDTQ487S-RBS55 was then cultivated under different shaking speeds, and the resulting 2-ketoisovalerate production and NADH level were compared (Fig. 5B and Fig. S4). At a shaking speed of 200 rpm, weakening the PDH in strain 050TY/pCTSDTQ487S-RBS55 resulted in significantly lower production of 2-ketoisovalerate than that of strain 050T4/pCTSDTQ487S-RBS55 (CK). Considering the weakening of a pyruvate-consuming route (PDH), the production should be significantly higher if no impact of redox balance was generated. These results indicated that the lower NADH accumulation in strain 050TY/pCTSDTQ487S-RBS55 (Fig. S4A) would not be sufficient for propelling 2-ketoisovalerate synthesis during aerobic cultivation. In contrast, the NADH/NAD^+^ ratio would be too high to be reoxidized through the ETC at the shaking speed of 50 rpm (Fig. S4B). The highest 2-ketoisovalerate titer and yields of the overall process, as well as the second phase, were achieved simultaneously under microaerobic cultivation at the shaking speed of 100 rpm (Fig. 5B), which were 1-, 1.2-, and 1.2-fold higher, respectively, than the aerobic results using strain 050T4/pCTSDT (Fig. 3C).

Lower cell mass was accumulated during microaerobic cultivation, which would be insufficient for 2-ketoisovalerate production, and therefore, the transition stage between aerobic growth and microaerobic production phases was further optimized (Fig. 5C). Increasing the aerobic cell mass to an OD_600_ of 4 significantly improved the 2-ketoisovalerate titer to 22 g/L. However, this level was significantly reduced if switched to the microaerobic production phase at an OD_600_ of 5, suggesting the impairment with overgrown cell cultures. Therefore, transition at an aerobic cell level of OD_600_ of 4 was the optimal condition.

Although 2-ketoisovalerate yield could also be improved by deleting the PDH-coding gene, acetate should be added as an extra carbon source for cell growth (6). In the present study, the PDH was dynamically switched to balance the processes of cell growth and 2-ketoisovalerate production without requiring acetate as a substrate. During the aerobic cell growth phase, the growth ability of strain 050TY/pCTSDTQ487S-RBS55 did not considerably reduce (Fig. 5B), which was consistent with the weaker degradation ability of the DAS+4 tag (Fig. S3). During the microaerobic 2-ketoisovalerate production phase, degrading the PDH with a protein degradation tag and inhibiting the expression and activity of PDH under microaerobic cultivation (38) successfully switched off the competing route and improved 2-ketoisovalerate yield. The lower reducing power generated through the inhibited PDH and TCA cycle (Fig. S4) required less O_2_ for the circulation of the redox carriers through the ETC. Redox balance was reached under microaerobic conditions, with the efficient production of 2-ketoisovalerate (Fig. 5C). As a result, high 2-ketoisovalerate yield and titer were achieved at the same time by coordinating the PDH and ETC in strain 050TY/pCTSDTQ487S-RBS55, which were improved by 18% and 15%, respectively, over strain 050T4/pCTSDT.

### 2-Ketoisovalerate fermentation in a 5-L bioreactor using strain 050TY/pCTSDTQ487S-RBS55.

**FIG 6:**
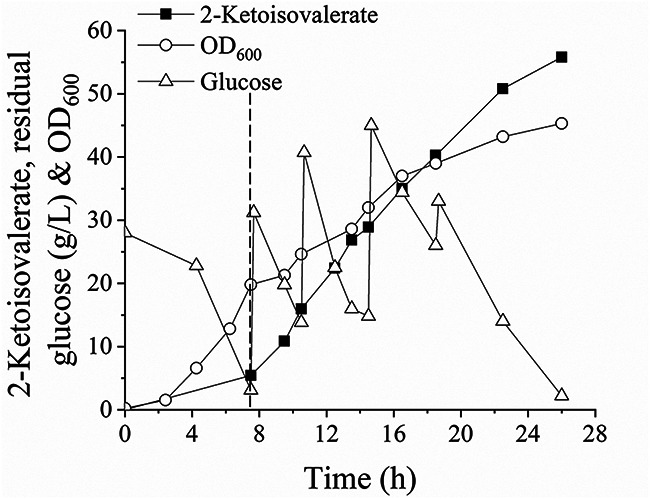
Representative experiment showing the production of 2-ketoisovalerate by strain 050TY/pCTSDTQ487S-RBS55 in a 5-L bioreactor. During the cell growth process, the dissolved O_2_ concentration was maintained above 30% saturation in a cascade by stirring and sparging air into the bioreactor. When the cell density reached an OD_600_ of approximately 20, the 2-ketoisovalerate production phase was initiated (indicated by the dotted line). During the second phase, the agitation was 400 rpm, and air sparging was 1 L/min. Additions of glucose of 60, 60, 60, and 30 g were made to the bioreactor to maintain the residual glucose concentration above 10 g/L.

Strain 050TY/pCTSDTQ487S-RBS55 was further tested for 2-ketoisovalerate production in a 5-L bioreactor using aerobic and microaerobic two-phase fermentation. According to the optimal cultivation conditions in the shake-flask experiment (Fig. 5B and C), the cell density for switching to the microaerobic 2-ketoisovalerate production phase was equally amplified at an OD_600_ of 20 (Fig. 6). As a result, 55.8 g/L 2-ketoisovalerate was produced. Compared to strain 050T4/pCTSDT, the 2-ketoisovalerate volumetric and specific productivities of strain 050TY/pCTSDTQ487S-RBS55 significantly increased by 20% and 45%, respectively, without an increase in glucose consumption rate (Table 1). Consequently, the 2-ketoisovalerate yield of the overall fermentation phase using strain 050TY/pCTSDTQ487S-RBS55 was 1.3-fold greater, and the yield of the second phase was improved to 0.992 mol/mol glucose, approaching the theoretical yield of 1 mol/mol glucose (Table 1). The accumulation of isobutanol was substantially reduced to 1.51 g/L, with a yield of 0.036 mol/mol glucose (Table 1), indicating the significance of engineering AlsS. The substantially lower succinate yield and cell density and higher acetate yield of strain 050TY/pCTSDTQ487S-RBS55 than that of strain 050T4/pCTSDT (Table 1, Fig. 6, and Fig. 3D) suggested the effect of switching off the TCA cycle under microaerobic conditions.

Several differences distinguish this work from previous efforts to accumulate 2-ketovalerate. First, 2-ketoisovalerate previously accumulates as a by-product of isobutanol (8), and the production of 2-ketoisovalerate and isobutanol as a mixture hampers their separation, purification, and application. This study represents the first work of producing 2-ketoisovalerate as the main product of E. coli. Second, the deletions of *leuA* and *ilvA* genes that are involved in the synthesis of branched-chain amino acids are seldom tested for the production of 2-ketoisovalerate (6, 9) or valine (39, 40). In the present study, the deletion of *leuA* could promote 2-ketoisovalerate production (Fig. 2A) and would also benefit the production of the downstream products. Third, the main by-product of isobutanol was effectively minimized by engineering the activity (34) and expression of the AlsS (Fig. 4). These results call for special attention to minimizing by-product through decreasing the promiscuous activity of the enzymes involved. Fourth, redox imbalance is generated during the synthesis of 2-ketoisovalerate, with two molecules of NADH accumulated and an extra molecule of NADPH required. Herein, tuning the supply of NADPH (Fig. 3A), limiting the TCA cycle through regulating O_2_ supply (Fig. 3B), and degrading PDH (Fig. 5) were combined to balance the intracellular redox power. These metabolic engineering strategies could be useful for the production of other chemicals with imbalanced redox status. Fifth, aerobic and microaerobic two-phase fermentation was applied for efficient 2-ketoisovalerate production (Fig. 6). During the aerobic cell growth phase, a high growth rate of strain 050TY/pCTSDTQ487S-RBS55 remained to obtain sufficient cell mass. During the microaerobic production phase, 2-ketoisovalerate production was switched on, and the PDH was switched off. The 2-ketoisovalerate titer, volumetric productivity, and yield of the overall fermentation phase were 1.6-, 2.7-, and 3.5-fold higher, respectively, than the previously aerobic fermentation (9). This fermentation engineering strategy is reported for the first time for 2-ketoisovalerate production and is also significantly different from that of isobutanol (generally produced under anaerobic cultivation).

In conclusion, deletion of the *ilvE* and *leuA* genes could promote 2-ketoisovalerate production, whereas the inactivation of *ilvA* adversely affected the 2-ketoisovalerate titer. The Q487S mutant of AlsS was effective in reducing the accumulation of isobutanol as the main by-product. Tuning the expression of the chromosomal *pntAB* gene with the strong T7 promoter could compensate for the NADPH deficiency and help to increase the 2-ketoisovalerate titer. Recycling the overaccumulated NADH under aerobic cultivation could achieve a higher 2-ketoisovalerate titer and productivity, although microaerobic conditions were beneficial for higher yields. Switching off PDH with a protein degradation tag and inhibiting the expression and activity under microaerobic conditions could effectively improve 2-ketoisovalerate yield. The bottleneck of redox imbalance could be relieved under microaerobic cultivation, attaining high flux for 2-ketoisovalerate production. The results of this investigation have revealed metabolic engineering strategies to improve the titer, productivity, and yield of a fermentative product with imbalanced redox status and have provided a foundation for the microbial production of 2-ketoisovalerate.

## MATERIALS AND METHODS

### Strains and plasmids.

The relevant characteristics of the E. coli strains and plasmids used in the present study are summarized in Table 2. Methods used to construct these strains and plasmids are described in the supplemental method. The relevant primers are provided in Table 3.

**TABLE 2:** E. coli strains and plasmids used in this study

Strain or plasmid	Relevant characteristics	Reference and/or source
Strains		
B0016-050	CICIM B0016, Δ*ack-pta* Δ*pflB* Δ*adhE* Δ*frdA* Δ*ldhA*	21
050T	B0016-050, *ilvE*::*T7RNAP*	This study
050T1	050T, Δ*ilvA*	This study
050T2	050T1, Δ*leuA*	This study
050T3	050T, Δ*leuA*	This study
050T4	050T3, P_pntA_ promoter::P_T7_ promoter	This study
050T4-1	050T3, P_pntA_ promoter::P_TM1_ promoter	This study
050T4-2	050T3, P_pntA_ promoter::P_TM3_ promoter	This study
050TY	050T4, infusion of the DAS+4 tag after the *aceF* gene	This study
Plasmids		
pSDC	*bla*, pETDuet plasmid harboring P_T7_-*alsS* (Bacillus subtilis)-P_T7_-*ilvD* (E. coli)-P_T7_-*ilvC* (E. coli) fragment	Maintained in our laboratory
pCSD	*bla*, pETDuet plasmid harboring P_T7_-*ilvC* (E. coli)-P_T7_-*alsS* (B. subtilis)-P_T7_-*ilvD* (E. coli) fragment	Maintained in our laboratory
pCDS	*bla*, pETDuet plasmid harboring P_T7_-*ilvC* (E. coli)-P_T7_-*ilvD* (E. coli)-P_T7_-*alsS* (B. subtilis) fragment	Maintained in our laboratory
pDSC	*bla*, pETDuet plasmid harboring P_T7_-*ilvD* (E. coli)-P_T7_-*alsS* (B. subtilis)-P_T7_-*ilvC* (E. coli) fragment	Maintained in our laboratory
pDCS	*bla*, pETDuet plasmid harboring P_T7_-*ilvD* (E. coli)-P_T7_-*ilvC* (E. coli)-P_T7_-*alsS* (B. subtilis) fragment	Maintained in our laboratory
pCTSDT	*bla*, pETDuet plasmid harboring P_T7_-*ilvC* (E. coli)-T7 terminator-P_T7_-*alsS* (B. subtilis)-P_T7_-*ilvD* (E. coli) fragment	Maintained in our laboratory
pCTSTDT	*bla*, pETDuet plasmid harboring P_T7_-*ilvC* (E. coli)-T7 terminator-P_T7_-*alsS* (B. subtilis)-T7 terminator-P_T7_-*ilvD* (E. coli) fragment	Maintained in our laboratory
pACYC-kan-das	*cm, kan*, DAS+4 tag-FRT-*kan*-FRT	Maintained in our laboratory
pHT-P3-sfgfp	*bla*, pHT plasmid harboring P3-*sfgfp* fragment	Maintained in our laboratory
pKD46	*bla*, γ-β*-exo* (red recombinase), temp-conditional pSC101 replicon	CGSC^*a*^, 42
pKD13	*bla*, *kan*, FRT-*kan*-FRT cassette	CGSC^*a*^, 42
pCP20	*bla*, FLP, temp-conditional replicon	CGSC^*a*^, 42
pPL451	*bla*, *cI*^ts^857, pR/pL promoter	43
pMD-*T7RNAP*	*bla*, *T7RNAP*; *T7RNAP* (PCR using P1/P2 primers) from E. coli BL21(DE3) cloned into pMD19-T vector	This study
pMD-*T7RNAP-kan*	*bla*, *kan*, *T7RNAP*-FRT-*kan*-FRT; FRT-*kan*-FRT cassette (PCR using P3/P4 primers and digested by *Eco*RI and *Sac*I) from pKD13 cloned into *Eco*RI and *Sac*I sites of pMD-*T7RNAP*	This study
pACYC-*pntAB*	*cm*, *pntAB*; *pntAB* gene (PCR using P17/P18 primers) from E. coli MG1655 cloned into reverse PCR fragment of pACYCDuet plasmid (using P19/P20 primers) through Gibson assembly	This study
pACYC-*kan-pntAB*	*cm*, *kan*, FRT-*kan*-FRT-P_T7_-*pntAB*; FRT-*kan*-FRT cassette (PCR using P21/P22 primers) from pKD13 cloned into reverse PCR fragment of pACYC-*pntAB* plasmid (using P23/P24 primers) through Gibson assembly	This study
pACYC-*kan-pntAB*-2	*cm*, *kan*, FRT-*kan*-FRT-P_T7_-*pntAB*; FRT-*kan*-FRT cassette; one-step PCR protocol based on the pACYC-*kan-pntAB* plasmid using P25/P26 primers	This study
pACYC-*kan*-T7	*cm*, *kan*, FRT-*kan*-FRT-P_T7_; one-step PCR protocol based on the pACYC-*kan-pntAB*-2 plasmid using P27/P28 primers	This study
pACYC-*kan*-TM1	*cm*, *kan*, FRT-*kan*-FRT-P_TM1_; one-step PCR protocol based on the pACYC-kan-*pntAB*-2 plasmid using P29/P30 primers	This study
pACYC-*kan*-TM3	*cm*, *kan*, FRT-*kan*-FRT-P_TM3_; one-step PCR protocol based on the pACYC-kan-*pntAB*-2 plasmid using P31/P32 primers	This study
pCTSDTQ424S	*bla*, pETDuet plasmid harboring P_T7_-*ilvC*-T7 terminator-P_T7_-*alsS*Q424S-P_T7_-*ilvD* fragment; one-step PCR protocol based on the pCTSDT plasmid using P37/P38 primers	This study
pCTSDTQ487S	*bla*, pETDuet plasmid harboring P_T7_-*ilvC*-T7 terminator-P_T7_-*alsS*Q487S-P_T7_-*ilvD* fragment; one-step PCR protocol based on the pCTSDT plasmid using P39/P40 primers	This study
pCTSDTQ488S	*bla*, pETDuet plasmid harboring P_T7_-*ilvC*-T7 terminator-P_T7_-*alsS*Q488S-P_T7_-*ilvD* fragment; one-step PCR protocol based on the pCTSDT plasmid using P41/P42 primers	This study
pCTSDTQ487S-RBS42	*bla*, pETDuet plasmid harboring P_T7_-*ilvC*-T7 terminator-P_T7_-RBS42-*alsS*Q424S-P_T7_-*ilvD* fragment; one-step PCR protocol based on the pCTSDTQ487S plasmid using P43/P44 primers	This study
pCTSDTQ487S-RBS55	*bla*, pETDuet plasmid harboring P_T7_-*ilvC*-T7 terminator-P_T7_-RBS55-*alsS*Q424S-P_T7_-*ilvD* fragment; one-step PCR protocol based on the pCTSDTQ487S plasmid using P45/P46 primers	This study
pPL-gfp-DAS+4	*bla*, pPL451 plasmid harboring *gfp-DAS+4* fragment; *gfp-DAS+4* fragment (PCR using P47/P48 primers) from pHT-P3-sfgfp cloned into reverse PCR fragment of pPL451 plasmid (using P49/P50 primers) through Gibson assembly	This study
pPL-gfp	*bla*, pPL451 plasmid harboring *gfp* fragment; *gfp* fragment (PCR using P47/P51 primers) from pHT-P3-sfgfp cloned into reverse PCR fragment of pPL451 plasmid (using P49/P52 primers) through Gibson assembly	This study

a Genetic Stock Center, Yale University.

**TABLE 3:** Primers used in this study

Primer	Sequence (5′–3′)^*a*^	Restriction site
P1	GCTTCCGGCTCGTATAATGTGTGG	
P2	TGCGCGCACGAAAAGCATCAGG	
P3	TTT*GAATTC*gtgtaggctggagctgcttc	*Eco*RI
P4	TTT*GAGCTC*attccggggatccgtcgacc	*Sac*I
P5	GACGGTGCGTGCCGTCCCATTTTTTGTATTTATTGATTAACTTGATCTAAgtgtaggctggagctgcttc	
P6	AACCACATCACAACAAATCCGCGCCTGAGCGCAAAAGGAATATAAAAATGGCTTCCGGCTCGTATAATGTGTGG	
P7	TCAATGAATATGGCCGCCG	
P8	ATCGGCGTCGGTCATTCC	
P9	GCCGACAAAGGCGCGGTGCGCGATAAATCGAAACTGGGGGGTTAATAATGgtgtaggctggagctgcttc	
P10	ATAAGCGAAGCGCTATCAGGCATTTTTCCCTAACCCGCCAAAAAGAACCTattccggggatccgtcgacc	
P11	TTATGCCAGCCTGGCAAC	
P12	ATTTTGCCGAACCACAAATG	
P13	CAATACGGCAATATGGTAATTCTTCGACATCACACGGTTTCCTTGTTGTTgtgtaggctggagctgcttc	
P14	TTTTATGCCCGAAGCGAGGCGCTCTAAAAGAGACAAGGACCCAAACCATGattccggggatccgtcgacc	
P15	TGGGTCATCACTTCCGGAC	
P16	TAAGCCAGCACGCAGTC	
P17	CATCACCACAGCCAGGATCCATGCGAATTGGCATACCAAG	
P18	CGCGCCGAGCTCGAATTCTTACAGAGCTTTCAGGATTGCATCC	
P19	GAATTCGAGCTCGGCGCG	
P20	GGATCCTGGCTGTGGTGATG	
P21	GCGACTCCTGCATTAGGAAATGTGTAGGCTGGAGCTGCTTC	
P22	TCCCCTATAGTGAGTCGTATTAATTCCGGGGATCCGTCGACCT	
P23	ATTTCCTAATGCAGGAGTCGC	
P24	TAATACGACTCACTATAGGGGACCTGTAGAAAT	
P25	CTGAAAGCTCTGTAATAATTAACCTAGGCTGCTGCCACCGC	
P26	TTACAGAGCTTTCAGGATTGCATCCACGC	
P27	CCTATAGTGAGTCGTATTAattccggggatccgtcgacc	
P28	TAATACGACTCACTATAGGGGA	
P29	CCATTAGTGAGTCGTATTAattccggggatccgtcgacc	
P30	TAATACGACTCACTAATGGGGA	
P31	TTTATAGTGAGTCGTATTAattccggggatccgtcgacc	
P32	TAATACGACTCACTATAAAGGA	
P33	TATTTTAACGGAGTAACATTTAGCTCGTACATGAGCAGCTTGTGTGGCTCgtgtaggctggagctgcttc	
P34	TTGGTTAACCGTTCTCTTGGTATGCCAATTCGCATGATATTCCCTTCCATGCTGCTGCCCATGGTATATCTCCTTATT	
P35	GCAACACGGGTTTCATTGG	
P36	CAGCATCATTACTACTGAAGCAACT	
P37	TCAGTAACGGTATGTCTACACTCGGCGTTGCGCTT	
P38	CAACGCCGAGTGTAGACATACCGTTACTGATCATTAATGTTAACGGCTCG	
P39	CATGGTTGCATTCTCTCAATTGAAAAAATATAACCGTACATCTGCGGTCG	
P40	ATATTTTTTCAATTGAGAGAATGCAACCATGTCATATGTGCTG	
P41	CATGGTTGCATTCCAGTCTTTGAAAAAATATAACCGTACATCTGCGGTCG	
P42	ATATTTTTTCAAAGACTGGAATGCAACCATGTCATATGTGCTG	
P43	AACCAAAGGAGGTTTAAATGTTAACAAAAGCAACAAAAGAACAAAAATCCCTTGTG	
P44	TTAAACCTCCTTTGGTTTTGGATACCCCTTAAAGTTAAACAAAATTATTTCTAGAGGGGAATTGTTATCCGC	
P45	AAATCTACTAAGGAGGCTATTAATGTTAACAAAAGCAACAAAAGAACAAAAATCCCTTGTG	
P46	TAATAGCCTCCTTAGTAGATTTCTCGAGCCTTAAAGTTAAACAAAATTATTTCTAGAGGGGAATTGTTATCCGC	
P47	TAAGGAGGTTAACTATGAGCAAAGGAGAAGAACTTTTCACT	
P48	GTAATTTTCGCTGTAATTTTCATCATTAGCTGCTTTGTAGAGCTCATCCATGCCATGTG	
P49	CATAGTTAACCTCCTTAGGATCCCAATGC	
P50	ATTACAGCGAAAATTACGCAGATGCCAGCTAATGGCCGTCGTTTTACAACGTCG	
P51	AACGACGGCCATTATTTGTAGAGCTCATCCATGCCA	
P52	CTACAAATAATGGCCGTCGTTTTACAACGTC	
P53	TCATTACCATCATTAACAACACGCTGTCTGACATTCGCCGTCTGGTGATGGCAGCTAATGATGAAAATTACAGCGAAAATTACG	
P54	CATTCATGAGATTACCAGAAAAAAGCCGGCCGTTGGGCCGGCTCTTTTACTgtgtaggctggagctgcttc	
P55	GGTTGCTTCACCATCTCCAGCATC	
P56	GTACCACGACCTGAGTTTTGATTTCAG	

a Restriction sites are indicated in italics. Underlined sequences indicate the homologous sequence with the E. coli genome. Lowercase letters indicate the homologous sequence with the pKD13 plasmid.

### Media.

Lysogeny broth (LB) medium was used for cell precultivation. The medium for shake-flask fermentation contained (per L) 6 g Na_2_HPO_4_, 3 g KH_2_PO_4_, 1 g NH_4_Cl, 0.3 g NaCl, and 5 g yeast extract. Briefly, 60 mL of 600 g/L glucose, 2 mL of filter-sterilized 1 M MgSO_4_, and 1 mL of filter-sterilized trace element solution containing (per L) 10 g FeSO_4_·7H_2_O, 3 g CuSO_4_·5H_2_O, 0.5 g MnSO_4_·4H_2_O, 5.25 g ZnSO_4_·7H_2_O, 0.1 g (NH_4_)Mo_7_O_24_, 0.2 g Na_2_B_4_O_7_·10H_2_O, and 2 g CaCl_2_ were added to 1 L of the final medium. The medium for bioreactor fermentation contained (per L) 4 g (NH_4_)_2_HPO_4_, 13.5 g KH_2_PO_4_, 1.7 g citric acid monohydrate, 4 g yeast extract, 4 g tryptone, 2 mL of filter-sterilized 1 M MgSO_4_, and 1 mL of filter-sterilized trace element solution. Ampicillin, kanamycin, and chloramphenicol were added to the media as needed to final concentrations of 100, 50, and 35 μg/mL, respectively.

### Shake-flask experiments.

To identify the properties of 2-ketoisovalerate production, the recombinant strains (stored as glycerol stocks at −80°C) were first grown on LB medium plates at 37°C for approximately 24 h, and subsequently, colonies were transferred to 50 mL of LB medium in a 250-mL flask. After 10 h of growth at 37°C with shaking at 200 rpm, the culture broth was transferred to 50 mL fresh medium containing 36 g/L glucose at an inoculation rate of 1% (vol/vol) to start a two-phase fermentation. All flasks were incubated at 37°C with shaking at 200 rpm during the initial cell growth phase. When the cell concentration reached the indicated level (OD_600_ of 0.8, 1.5, 2.5, 4, or 5), isopropyl β-d-1-thiogalactoside (IPTG) was added to the broth to a final concentration of 0.4 mM. The flasks were kept at 30°C with shaking at the indicated speed (0, 50, 100, 150, or 200 rpm) to start the 2-ketoisovalerate production phase, and concentrated NH_4_OH was added every 4 h to maintain the pH at 7.

### Bioreactor experiments.

For each bioreactor experiment, cells were precultured in LB medium, as described in the shake-flask experiments. The 100-mL LB broth was used as a seed culture to inoculate 2-L medium with 30 g/L glucose, which was contained in a 5-L bioreactor (Winpact FS-02; Major Science, Saratoga, CA, USA). A two-phase fermentation with a cell growth phase was maintained at 37°C, and then a 2-ketoisovalerate production phase maintained at 30°C was initiated. Dissolved O_2_ concentration was controlled at the indicated saturation by stirring at 200 to 1,000 rpm and sparging air into the bioreactor at 3 to 10 L/min. To adjust the pH to 7, concentrated NH_4_OH was added automatically. At the initiation of the second phase, 0.38 g IPTG was added to the broth. Six batches of 5 g yeast extract and 5 g peptone were fed into the bioreactor as an organic nitrogen source to obtain a higher cell density. Glucose was fed into the bioreactor to maintain the residual glucose concentration above 10 g/L.

### Analytical methods.

The cell mass was determined by measuring the optical density at 600 nm. Glucose concentration was measured using a glucose biosensor (SBA-40E; Biology Institute of Shandong Academy of Sciences, Tsinan, China) (21). 2-Ketoisovalerate in the cell culture supernatant was measured by high-pressure liquid chromatography (HPLC) equipped with UV (210 nm) using a Prevail organic acid column (250 by 4.6 mm, 5 μm) (Grace Davison Discovery Sciences, Columbia, MD, USA) with 25 mM KH_2_PO_4_ (pH 2.5) as eluent (1 mL/min; 40°C). Isobutanol in the cell culture supernatant was assayed by gas chromatography-mass spectrometry (GC-MS) as described previously (41). The activity of PntAB was assayed using the transhydrogenase-1 (TH-1) assay kit (ZCIBIO Technology, Shanghai, China). The activity of UdhA was assayed using the transhydrogenase-2 (TH-2) assay kit (Beijing Solarbio Science & Technology, Beijing, China). The activity of PDH was detected using the PDH activity assay kit with colorimetry (D799371-0050; Sangon Biotechnology, Shanghai, China) according to the manufacturer’s instructions. The intracellular ratio of NADPH/NADP^+^ was assayed using the NADP^+^/NADPH assay kit with WST-8 (Beyotime Biotechnology, Shanghai, China). Intracellular levels of NADH and NAD^+^ were assayed using the NAD^+^/NADH assay kit with WST-8 (Beyotime Biotechnology). Standard deviations were calculated to show the parallelism of the experiments as indicated by the error bars. *P* values of <0.05 were considered statistically significant, and statistical significance is indicated as *, *P < *0.05; **, *P < *0.01; and ***, *P < *0.001.

## References

[1] Aparicio M, Cano NJ, Cupisti A, Ecder T, Fouque D, Garneata L, Liou HH, Lin S, Schober-Halstenberg HJ, Teplan V, Zakar G. 2009. Keto-acid therapy in predialysis chronic kidney disease patients: consensus statements. J Ren Nutr 19:S33–5. 10.1053/j.jrn.2009.06.013. 19712876

[2] Chang JH, Kim DK, Park JT, Kang EW, Yoo TH, Kim BS, Choi KH, Lee HY, Han DS, Shin SK. 2009. Influence of ketoanalogs supplementation on the progression in chronic kidney disease patients who had training on low-protein diet. Nephrology (Carlton) 14:750–757. 10.1111/j.1440-1797.2009.01115.x. 20025684

[3] Schaefer K, von Herrath D, Erley CM, Asmus G. 1990. Calcium ketovaline as new therapy for uremic hyperphosphatemia. Miner Electrolyte Metab 16:362–364. 2089249

[4] Lu J, Brigham CJ, Plassmeier JK, Sinskey AJ. 2015. Characterization and modification of enzymes in the 2-ketoisovalerate biosynthesis pathway of *Ralstonia eutropha* H16. Appl Microbiol Biotechnol 99:761–774. 10.1007/s00253-014-5965-3. 25081555

[5] Atsumi S, Li Z, Liao JC. 2009. Acetolactate synthase from *Bacillus subtilis* serves as a 2-ketoisovalerate decarboxylase for isobutanol biosynthesis in *Escherichia coli*. Appl Environ Microbiol 75:6306–6311. 10.1128/AEM.01160-09. 19684168 PMC2753059

[6] Krause FS, Blombach B, Eikmanns BJ. 2010. Metabolic engineering of *Corynebacterium glutamicum* for 2-ketoisovalerate production. Appl Environ Microbiol 76:8053–8061. 10.1128/AEM.01710-10. 20935122 PMC3008247

[7] Cooper AJL, Ginos JZ, Meister A. 1983. Synthesis and properties of the alpha-keto acids. Chem Rev 83:38. 10.1021/cr00055a004.

[8] Gu J, Zhou J, Zhang Z, Kim CH, Jiang B, Shi J, Hao J. 2017. Isobutanol and 2-ketoisovalerate production by *Klebsiella pneumoniae* via a native pathway. Metab Eng 43:71–84. 10.1016/j.ymben.2017.07.003. 28802880

[9] Buchholz J, Schwentner A, Brunnenkan B, Gabris C, Grimm S, Gerstmeir R, Takors R, Eikmanns BJ, Blombach B. 2013. Platform engineering of *Corynebacterium glutamicum* with reduced pyruvate dehydrogenase complex activity for improved production of L-lysine, L-valine, and 2-ketoisovalerate. Appl Environ Microbiol 79:5566–5575. 10.1128/AEM.01741-13. 23835179 PMC3754147

[10] Klamt S, Mahadevan R, Hadicke O. 2018. When do two-stage processes outperform one-stage processes? Biotechnol J 13. 10.1002/biot.201700539. 29131522

[11] Choi KR, Jang WD, Yang D, Cho JS, Park D, Lee SY. 2019. Systems metabolic engineering strategies: integrating systems and synthetic biology with metabolic engineering. Trends Biotechnol 37:817–837. 10.1016/j.tibtech.2019.01.003. 30737009

[12] Zhou L, Niu DD, Tian KM, Chen XZ, Prior BA, Shen W, Shi GY, Singh S, Wang ZX. 2012. Genetically switched D-lactate production in *Escherichia coli*. Metab Eng 14:560–568. 10.1016/j.ymben.2012.05.004. 22683845

[13] Chen X, Li S, Liu L. 2014. Engineering redox balance through cofactor systems. Trends Biotechnol 32:337–343. 10.1016/j.tibtech.2014.04.003. 24794722

[14] Bastian S, Liu X, Meyerowitz JT, Snow CD, Chen MM, Arnold FH. 2011. Engineered ketol-acid reductoisomerase and alcohol dehydrogenase enable anaerobic 2-methylpropan-1-ol production at theoretical yield in *Escherichia coli*. Metab Eng 13:345–352. 10.1016/j.ymben.2011.02.004. 21515217

[15] Zhan M, Kan B, Dong J, Xu G, Han R, Ni Y. 2019. Metabolic engineering of *Corynebacterium glutamicum* for improved L-arginine synthesis by enhancing NADPH supply. J Ind Microbiol Biotechnol 46:45–54. 10.1007/s10295-018-2103-8. 30446890

[16] Jung HR, Yang SY, Moon YM, Choi TR, Song HS, Bhatia SK, Gurav R, Kim EJ, Kim BG, Yang YH. 2019. Construction of efficient platform *Escherichia coli* strains for polyhydroxyalkanoate production by engineering branched pathway. Polymers 11:509. 10.3390/polym11030509. PMC6473851 30960493

[17] Choi YJ, Lee J, Jang YS, Lee SY. 2014. Metabolic engineering of microorganisms for the production of higher alcohols. mBio 5:e01524-14. 10.1128/mBio.01524-14. 25182323 PMC4173780

[18] Zhou L, Tian KM, Niu DD, Shen W, Shi GY, Singh S, Wang ZX. 2012. Improvement of D-lactate productivity in recombinant *Escherichia coli* by coupling production with growth. Biotechnol Lett 34:1123–1130. 10.1007/s10529-012-0883-x. 22367280

[19] Li Y, Li M, Zhang X, Yang P, Liang Q, Qi Q. 2013. A novel whole-phase succinate fermentation strategy with high volumetric productivity in engineered *Escherichia coli*. Bioresour Technol 149:333–340. 10.1016/j.biortech.2013.09.077. 24125798

[20] Smith KM, Liao JC. 2011. An evolutionary strategy for isobutanol production strain development in *Escherichia coli*. Metab Eng 13:674–681. 10.1016/j.ymben.2011.08.004. 21911074

[21] Zhou L, Deng C, Cui WJ, Liu ZM, Zhou ZM. 2016. Efficient L-alanine production by a thermo-regulated switch in *Escherichia coli*. Appl Biochem Biotechnol 178:324–337. 10.1007/s12010-015-1874-x. 26453031

[22] Holatko J, Elisakova V, Prouza M, Sobotka M, Nesvera J, Patek M. 2009. Metabolic engineering of the L-valine biosynthesis pathway in *Corynebacterium glutamicum* using promoter activity modulation. J Biotechnol 139:203–210. 10.1016/j.jbiotec.2008.12.005. 19121344

[23] McCourt JA, Duggleby RG. 2006. Acetohydroxyacid synthase and its role in the biosynthetic pathway for branched-chain amino acids. Amino Acids 31:173–210. 10.1007/s00726-005-0297-3. 16699828

[24] Westbrook AW, Ren X, Moo-Young M, Chou CP. 2018. Metabolic engineering of *Bacillus subtilis* for l-valine overproduction. Biotechnol Bioeng 115:2778–2792. 10.1002/bit.26789. 29981237

[25] Li Y, Zhou L, Zhou Z. 2020. Three enzyme coupling to synthesize α-ketoisovalerate in *E. coli*. Food Ferment Ind 46:18–23. (In Chinese.) 10.13995/j.cnki.11-1802/ts.022717.

[26] Hasegawa S, Jojima T, Suda M, Inui M. 2020. Isobutanol production in *Corynebacterium glutamicum*: suppressed succinate by-production by *pckA* inactivation and enhanced productivity *via* the Entner–Doudoroff pathway. Metab Eng 59:24–35. 10.1016/j.ymben.2020.01.004. 31926306

[27] Mairhofer J, Wittwer A, Cserjan-Puschmann M, Striedner G. 2015. Preventing T7 RNA polymerase read-through transcription—a synthetic termination signal capable of improving bioprocess stability. ACS Synth Biol 4:265–273. 10.1021/sb5000115. 24847676

[28] Sauer U, Canonaco F, Heri S, Perrenoud A, Fischer E. 2004. The soluble and membrane-bound transhydrogenases UdhA and PntAB have divergent functions in NADPH metabolism of *Escherichia coli*. J Biol Chem 279:6613–6619. 10.1074/jbc.M311657200. 14660605

[29] Zhang C, Seow VY, Chen X, Too HP. 2018. Multidimensional heuristic process for high-yield production of astaxanthin and fragrance molecules in *Escherichia coli*. Nat Commun 9:1858. 10.1038/s41467-018-04211-x. 29752432 PMC5948211

[30] Ou B, Garcia C, Wang Y, Zhang W, Zhu G. 2018. Techniques for chromosomal integration and expression optimization in *Escherichia coli*. Biotechnol Bioeng 115:2467–2478. 10.1002/bit.26790. 29981268

[31] Alper H, Fischer C, Nevoigt E, Stephanopoulos G. 2005. Tuning genetic control through promoter engineering. Proc Natl Acad Sci USA 102:12678–12683. 10.1073/pnas.0504604102. 16123130 PMC1200280

[32] Zelcbuch L, Lindner SN, Zegman Y, Vainberg Slutskin I, Antonovsky N, Gleizer S, Milo R, Bar-Even A. 2016. Pyruvate formate-lyase enables efficient growth of *Escherichia coli* on acetate and formate. Biochemistry 55:2423–2426. 10.1021/acs.biochem.6b00184. 27093333

[33] Wang X, Wang A, Zhu L, Hua D, Qin J. 2018. Altering the sensitivity of *Escherichia coli* pyruvate dehydrogenase complex to NADH inhibition by structure-guided design. Enzyme Microb Technol 119:52–57. 10.1016/j.enzmictec.2018.09.002. 30243387

[34] Sommer B, von Moeller H, Haack M, Qoura F, Langner C, Bourenkov G, Garbe D, Loll B, Bruck T. 2015. Detailed structure–function correlations of *Bacillus subtilis* acetolactate synthase. Chembiochem 16:110–118. 10.1002/cbic.201402541. 25393087

[35] Salis HM, Mirsky EA, Voigt CA. 2009. Automated design of synthetic ribosome binding sites to control protein expression. Nat Biotechnol 27:946–950. 10.1038/nbt.1568. 19801975 PMC2782888

[36] Hao Y, Ma Q, Liu X, Fan X, Men J, Wu H, Jiang S, Tian D, Xiong B, Xie X. 2020. High-yield production of L-valine in engineered *Escherichia coli* by a novel two-stage fermentation. Metab Eng 62:198–206. 10.1016/j.ymben.2020.09.007. 32961297

[37] McGinness KE, Baker TA, Sauer RT. 2006. Engineering controllable protein degradation. Mol Cell 22:701–707. 10.1016/j.molcel.2006.04.027. 16762842

[38] Partridge JD, Sanguinetti G, Dibden DP, Roberts RE, Poole RK, Green J. 2007. Transition of *Escherichia coli* from aerobic to micro-aerobic conditions involves fast and slow reacting regulatory components. J Biol Chem 282:11230–11237. 10.1074/jbc.M700728200. 17307737

[39] Hao Y, Pan X, Xing R, You J, Hu M, Liu Z, Li X, Xu M, Rao Z. 2022. High-level production of L-valine in *Escherichia coli* using multi-modular engineering. Bioresour Technol 359:127461. 10.1016/j.biortech.2022.127461. 35700900

[40] Geraskina NV, Sycheva EV, Samsonov VV, Eremina NS, Hook CD, Serebrianyi VA, Stoynova NV. 2019. Engineering *Escherichia coli* for autoinducible production of L-valine: an example of an artificial positive feedback loop in amino acid biosynthesis. PLoS One 14:e0215777. 10.1371/journal.pone.0215777. 31022249 PMC6483228

[41] Atsumi S, Hanai T, Liao JC. 2008. Non-fermentative pathways for synthesis of branched-chain higher alcohols as biofuels. Nature 451:86–89. 10.1038/nature06450. 18172501

[42] Datsenko KA, Wanner BL. 2000. One-step inactivation of chromosomal genes in *Escherichia coli* K-12 using PCR products. Proc Natl Acad Sci USA 97:6640–6645. 10.1073/pnas.120163297. 10829079 PMC18686

[43] Love CA, Lilley PE, Dixon NE. 1996. Stable high-copy-number bacteriophage lambda promoter vectors for overproduction of proteins in *Escherichia coli*. Gene 176:49–53. 10.1016/0378-1119(96)00208-9. 8918231

