# Supplementary material for: Evaluation of Metabolic Engineering Strategies on 2-Ketoisovalerate Production by Escherichia coli
Source: Appl Environ Microbiol. 2022 Aug 18;88(17):e00976-22. doi: 10.1128/aem.00976-22 (PMC9469723; doi:10.1128/aem.00976-22)
Supplement: Supplemental file 1 — Supplemental text and Fig. S1 to S4. Download aem.00976-22-s0001.pdf, PDF file, 0.6 MB [file aem.00976-22-s0001.pdf]

Evaluation of metabolic engineering strategies on 2-ketoisovalerate production by *Escherichia coli*

Li Zhou,[a] Ying Zhu,[a] Zhongzhe Yuan,[a] Guangqing Liu,[a] Zijin Sun,[a] Shiyu Du,[a] He Liu,[a] Yating Li,[a] Haili Liu,[a] Zhemin Zhou[a] #

[a]The Key Laboratory of Industrial Biotechnology of Ministry of Education, School of Biotechnology, Jiangnan University, Wuxi, People's Republic of China

Running Head: 2-Ketoisovalerate production strategy by *E. coli*

Adress correspondence to Zhemin Zhou, zhmzhou@jiangnan.edu.cn

School of Biotechnology, Jiangnan University, 1800 Lihu Avenue, Wuxi 214122, People's Republic of China.

SUPPLEMENTARY METHOD

Chromosomal gene modification was conducted as described by Datsenko et al. (1), and all the resultant strains were confirmed by PCR and DNA sequencing. The *Escherichia coli* strains 050T, 050T1, 050T2, 050T3 were constructed by serial gene deletions in *E. coli* CICIM B0016-050 (*ack*-*pta pflB adhE frdA ldhA*) (2). In order to obtain strain 050T, recombinant plasmid pMD-*T7RNAP* was constructed first by cloning T7 RNA polymerase gene (*T7RNAP*) from *E. coli* BL21 (DE3) (using primers P1 and P2) into pMD19-T vector. Subsequently, the FRT-*kan*-FRT cassette that PCR amplified using P3 and P4 primers from pKD13 plasmid was cloned into the *Eco*RI and *Sac*I sites of pMD-*T7RNAP* to generate plasmid pMD-*T7RNAP-kan*. The *T7RNAP*-FRT-*kan*-FRT fragment was amplified from pMD-*T7RNAP-kan* using the primers P5 and P6 and was used to replace the chromosomal *ilvE* gene in strain B0016-050 according to the previous report (1). The resulting strain 050T was verified by PCR amplification using primers P7 and P8. To construct strain 050T1, the FRT-*kan*-FRT cassette amplified from pKD13 plasmid with primers P9 and P10 was used to replace the chromosomal *ilvA* gene in strain 050T, and the resulting strain was verified by PCR amplification using primers P11 and P12. In order to delete the chromosomal *leuA* gene in strain 050T1 and 050T, the FRT-*kan*-FRT cassette was amplified with the primers P13 and P14, and the resulting strains 050T2 and 050T3 were confirmed by PCR using primers P15 and P16.

To regulate the expression of pyridine nucleotide transhydrogenase (encoded by *pntAB* gene), relevant over-expression plasmid and strain were constructed. The structural region of *pntAB* gene was cloned from the genomic DNA of *E. coli* MG1655 using primers P17 and P18. This PCR fragment was cloned into the reverse PCR fragment that was amplified by P19 and P20 primers from pACYCDuet plasmid through Gibson assembly, to create pACYC-*pntAB*. To replace the chromosomal P_{pntA} promoter, the selection marker cassette FRT-*kan*-FRT (amplified by P21 and P22 primers from pKD13 plasmid) was cloned into the reverse PCR fragment amplified using P23 and P24 primers from pACYC-*pntAB* plasmid through Gibson assembly, and plasmid pACYC-*kan-pntAB* was created. The second T7 promoter on pACYC-*kan-pntAB* plasmid was deleted through one-step PCR using primers P25 and P26 to produce pACYC-*kan-pntAB*-2. Thereafter, plasmids pACYC-*kan*-T7, pACYC-*kan*-TM1 and pACYC-*kan*-TM3 with varied promoter strength were constructed through one-step PCR protocol based on the pACYC-*kan-pntAB*-2 plasmid using P27/P28, P29/P30 and P31/P32 primers, respectively. The PCR fragments amplified from pACYC-*kan*-T7, pACYC-*kan*-TM1 and pACYC-*kan*-TM3 plasmids using P33 and P34 primers were used to substitute the chromosomal P_{pntA} promoter in strain 050T3 to create 050T4, 050T4-1 and 050T4-2, respectively. These recombinant strains were verified by PCR using P35 and P36 primers.

To reduce the accumulation of isobutanol, the *alsS* gene was modified. Plasmids pCTSDTQ424S, pCTSDTQ487S and pCTSDTQ488S were constructed through one-step PCR protocol based on plasmid pCTSDT using P37/P38, P39/P40 and P41/P42 primers, respectively. The RBS of *alsS* gene on pCTSDTQ487S plasmid was modified through one-step PCR protocol using P43/P44 and P45/P46 primers to construct plasmids pCTSDTQ487S-RBS42 and pCTSDTQ487S-RBS55, respectively.

To promote degradation of pyruvate dehydrogenase complex, the DNA fragments harboring the DAS+4 degradation tag (3) and the selection marker cassette FRT-*kan*-FRT was amplified from plasmid pACYC-kan-das, using primers P53/P54. This fragment was inserted after the chromosomal *aceF* gene in strain 050T4, to create 050TY. The resulting strains were verified by P55/P56 primers.

The recombinant plasmids pSDC, pCSD, pCDS, pDSC, pDCS, pCTSDT, pCTSTDT (maintained in our laboratory), pCTSDTQ424S, pCTSDTQ487S, pCTSDTQ488S, pCTSDTQ487S-RBS42, pCTSDTQ487S-RBS55 and pACYC-pntAB (for over-expression of *alsS*, *ilvC*, *ilvD* or *pntAB* genes) were transformed into the above chromosomally modified strains for the investigation of 2-ketoisovalerate fermentation.

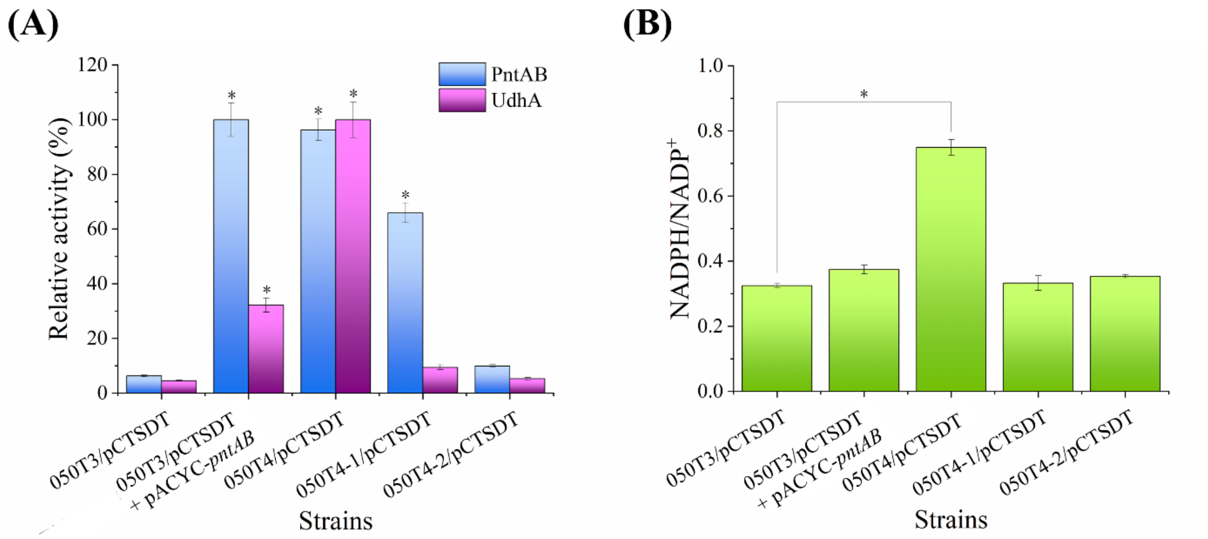

FIG S1 Effects of overexpressing *pntAB*. (A) Relative activities of PntAB and UdhA. (B) $NADPH/NADP^+$ ratio. Strains were induced by IPTG for 4 h.

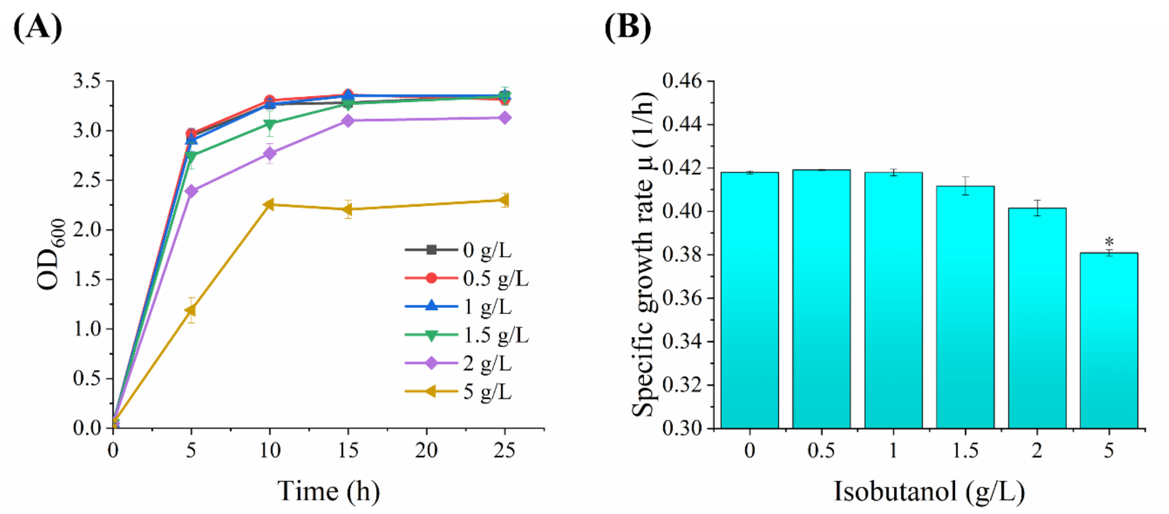

FIG S2 The impact of isobutanol concentration on the growth of strain 050T4. (A) The growth of strain 050T4 with isobutanol supplementation. (B) The specific growth rate of strain 050T4 with isobutanol supplementation.

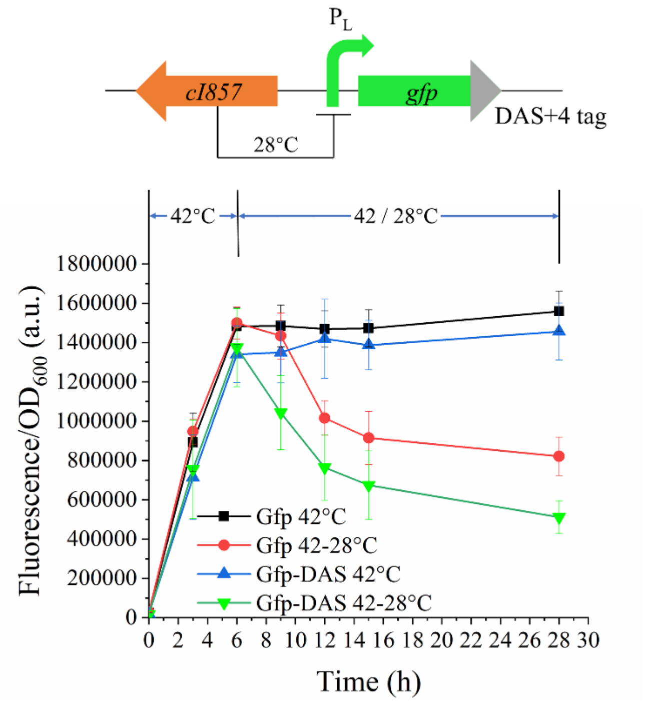

FIG S3 Verification of the DAS+4 tag in strain *E. coli* 050T4. The 050T4/pPL-gfp and 050T4/pPL-gfp-DAS+4 strains were initially cultivated at 42°C for 6 h, then at 42 or 28°C for 22 h.

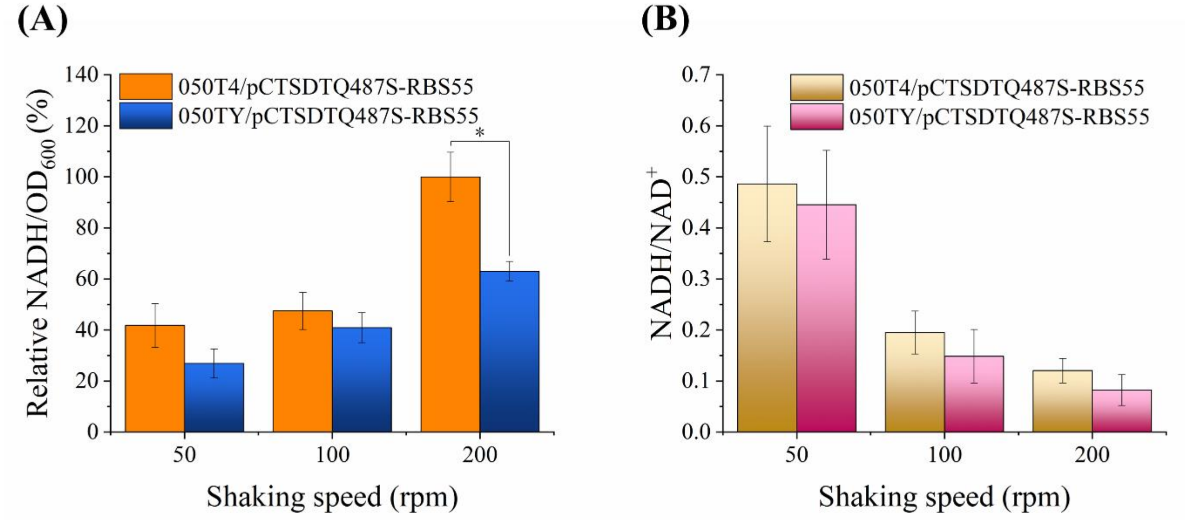

FIG S4 Effects of shaking speed on redox level in strains 050T4/pCTSDTQ487S-RBS55 and 050TY/pCTSDTQ487S-RBS55. (A) Relative NADH/OD_{600}. (B) NADH/NAD^+ ratio. Strains were cultured at the indicated shaking speed for 4 h.

SUPPLEMENTARY REFERENCES

1. Datsenko KA, Wanner BL. 2000. One-step inactivation of chromosomal genes in *Escherichia coli* K-12 using PCR products. Proc Natl Acad Sci U S A 97:6640-5.

2. Zhou L, Deng C, Cui WJ, Liu ZM, Zhou ZM. 2016. Efficient L-alanine production by a thermo-regulated switch in *Escherichia coli*. Appl Biochem Biotechnol 178:324-37.

3. McGinness KE, Baker TA, Sauer RT. 2006. Engineering controllable protein degradation. Mol Cell 22:701-7.
